# *Quercus infectoria* gall extracts reduce quorum sensing-controlled virulence factors production and biofilm formation in *Pseudomonas aeruginosa* recovered from burn wounds

**DOI:** 10.1186/s12906-019-2594-5

**Published:** 2019-07-18

**Authors:** Akhter Ahmed Ahmed, Fraidoon Abdulqadir Salih

**Affiliations:** Department of Biology College of Science, Salahaddin University-Erbil, Erbil, Kurdistan Region Iraq

**Keywords:** *Pseudomonas aeruginosa*, Quorum sensing, Inhibitor, Exotoxin A, *Quercus infectoria*

## Abstract

**Background:**

*Quercus* gall extracts’ ability to kill pathogens in vitro and even removal of chronic drug-resistant infections has been reported by several studies. The current investigation is focused on the action of extracts of *Quercus infectoria* gall in their sub-inhibitory concentrations on the corresponding bacterial behaviours instead of killing them.

**Methods:**

The effect of gall extracts on the quorum sensing (QS) associated virulence of multiple drug resistant *Pseudomonas aeruginosa* recovered from burns wounds was studied. The influence of different extracts on the production of bacterial virulence and biofilm, and expression of the genes encoding quorum sensing and exotoxin A were investigated. Quorum sensing is a crucial regulator of virulence and biofilm development in *Pseudomonas aeruginosa* and other medical related microbes.

**Results:**

Experiments to characterise and quantify *Q. infectoria* gall extracts impact on the quorum sensing networks of *P.aeruginosa* revealed that the expression of las, rhl, and exotoxin A (*ETA*) genes levels including the associated virulence were reduced by the extracts at their subinhibitory concentrations.

**Conclusions:**

The obtained results indicated that extracts of *Q. infectoria* galls fight infections either by their inhibitory constituents, which vigorously eradicate cells or by disruption of the pathogens quorum sensing system through weakening the virulence and bacterial coordination.

## Background

The beginning of twentieth century brought relief to human beings from a substantial number of life-threatening illnesses through the invention of antibiotics. As the twenty-first century begins, the excessive and undistinguished use of antimicrobials has led to the occurrence of multiple-drug-resistant (MDR) strains [1]. The fact that infectious diseases caused by MDR strains kill 16 million people every year resulted the necessity for alternative approaches to fight these MDRs [2, 3]. The promising tactic to develop distinctive antipathogenic treatment is through inhibiting microbial coordination, which is known as quorum sensing (QS). The QS is a type of communication between bacterial cells based on the concentration of “autoinducers” (AIs) which are small mediating molecules produced during the bacterial growth and regulate the expression of particular genes [4]. When a concentration threshold is reached, these AIs bind to their cognate receptors to form an AI–receptor complex, which will bind to the target promoter that leads to regulation of QS genes particularly those related to virulence [5]. The most studied intra-species AIs are small post-transnationally processed peptides and N-acyl homoserine lactone (AHL) in Gram-positive and Gram-negative bacteria, respectively [3, 6, 7]. Bacterial QS system regulates a wide array of phenotypes and controls the expression of various sets of genes such as bioluminescence, pigment production, biofilm formation, and antibiotic production [8].

The ordinarily adaptive pathogen *P. aeruginosa*, a Gram-negative bacterium, causes many nosocomial acquired infections, including infected burns and the genetic disease cystic fibrosis, especially in immunocompromised individuals [9].

The two well-studied QS systems in *P. aeruginosa* are Las and Rhl, which composed of − 3-oxo-dodecanoyl homoserine lactone and N-butanoyl homoserine lactone as AIs, and their transcriptional regulators LasR and RhlR, respectively [10]. QS systems control numerous virulence factors of *P. aeruginosa* like production of ETA, LasA protease, pyocyanin, LasB elastase, alkaline protease, and biofilm development [11]. Substantial attempts have been made to attain substances that inhibit the function of QS systems. Halogenated furanones which extracted from the red algae (*Delisea pulchra*) was the first described anti-QS agent. Awkwardly, these furanones are excessively reactive, and due to their toxicity to human, characterisation of novel non-toxic substances having anti-QS activity is a critical necessity [12].

QS has become an intense target for the improvement of unique anti-infective means that do not depend on the use of antimicrobials, and anti-QS substances are able to proscribe bacterial pathogenicity. The use of natural products has protracted attention due to increasing concerns over the safety of synthetic chemicals and emerging bacterial resistance to antibiotics [13]. Medicinal plants compromise an attractive array of phytochemicals with novel potential to control microbial diseases. This is due to the variety of secondary metabolites extant in plant extracts, which include phenolics, alkaloids, flavonoids, quinones, terpenoids, and poly-acetylenes [14].

The *Q. infectoria* (Fagaceae), the oak tree is broadly disseminated through the Northern provinces of Iraq, and it is an essential source of fibers and wood [15]. The galls can be seen on young branches of the tree as abnormal growth initiated by an increase in the size (hypertrophy) or number (hyperplasia) of plant cells as a response to the attack of the gall-wasp *Adleria gallaetinctoria*. The galls have been shown numerous medicinal properties such as antifungal, antibacterial, antiviral, larvicidal, local anesthetic, antidiabetic, astringent, and anti-inflammatory effects [16, 17]. The main chemical contents of the galls have been described are tannins, free gallic acids, synergic, and ellagic acid [18, 19].

Previous researches have reported that *Q. infectoria* gall extracts can prevent or eradicate the growth of many pathogens [20]. So far few studies have assessed the anti-QS and virulence; in addition, the impact of the gall extracts on ETA of *P. aeruginosa* at the expression level has not investigated yet. To examine the role of *Q. infectoria* gall extracts in the weakening of the virulence factors of *P. aeruginosa* through reducing the expression of genes in the Las and Rhl networks this study was conducted.

## Methods

### Plant material and extraction

Galls of *Q. infectoria* were collected from the oak trees of Kurdistan in 2016 fall and identified by the Herbarium of Biology Department College of Science, Salahaddin University-Erbil, Iraq. Galls were processed by the methods described previously by Harbome [21]. Briefly, pulverised plant material (after cleaning and shade drying) was extracted by sonication using different solvents: ethyl acetate, n-butanol, ethanol, and water. All solvents used for the extraction process were used as blanks and controls in all experiments through the study to exclude the effect of the solvents. To remove the solvents from the extracts vacuum evaporator was used to attain the crude extract of each fraction. The extracts stored at -20 °C and freshly dissolved in 10% dimethyl sulfoxide (DMSO, Merk, Germany) prior use.

### Specimen’s collection and sources

Twenty-five non-duplicate isolates of *P. aeruginosa* were collected from burns swabs submitted to be tested for bacteriology from hospitalised patients admitted to the West Emergency Hospital Erbil City, Iraqi Kurdistan. The specimens were taken by a sterile cotton swab from exudates and ulcers of the burns. The swabs were initially cultured onto Cetrimide and MacConkey agar media (acumedia, Neogen, USA) and incubated for 24 h at 37 °C. The individual colonies were identified as *P. aeruginosa* by various biochemical and conventional diagnostic tests, as described previously [22]. To confirm the identification, Vitek II automated system (bioMérieux Marcy l’’Etoile, France) (Vitek Systems Version: 06.01) was used. To test their susceptibility, the identified isolates subjected to a set of antimicrobials (Amikacin, Ceftazidime, Chloramphenicol, Ciprofloxacin, Doxycycline, Meropenem, Netilmicin, and Tobramycin) by disc diffusion method and Vitek II automated system, then the most resistant isolate was selected for all experiments in the study. The distinct isolates were stored in 1 ml Tryptic Soy Broth (TSB) (Oxoid) supplemented with 30% glycerol at − 70 °C for further study.

### Determination of minimum inhibitory and bactericidal concentrations (MICs and MBCs)

To determine the MICs for the gall extracts against the identified multidrug-resistant *P. aeruginosa* isolates broth microdilution method was used [23]. Ten μl from stationary-phase *P. aeruginosa* cells (equilibrated to OD550 0.5) was inoculated in 100 μl Nutrient broth (NB; Oxoid, UK) in 96-polystyrene microtitre plates (MTPs) containing a range of extracts concentrations (1–30 mg ml^− 1^). After 24 h incubation, the MICs were determined as the lowest concentrations with no evident growth. To assess the MBCs, 100 μl from the wells with no apparent growth was spread on Nutrient agar (NA; Oxoid, UK) plates and incubated at 37 °C for 24 h. The MBCs were detected as the concentration where no growth was detected on NA. Three biological replicates were analysed on separate instances. The levels below the MICs were considered as subinhibitory concentrations (SICs) and were further used in the assessment of the anti-virulence, anti-biofilm activity, and gene expression in the isolates of *P.aeruginosa*.

### Growth and viability

The non-growth-inhibitory action of the plant extracts was confirmed by flask incubation assay [24] to emphasise the anti-QS potency. 1% from a 24 h culture (equilibrated to 0.5 at 550 nm) of the *P. aeruginosa* isolate was inoculated into 50 ml of Luria-Bertani (LB) medium containing the SICs of plant extracts in Erlenmeyer flasks. The inoculated flasks were incubated at 37 °C with 180 rpm agitation in a rotatory shaker for 24 h, and the OD550 was monitored at hourly interims. Uninoculated flasks were used as blanks for plants extracts concentrations, and three biological replicates were analysed. The impact of extracts on the bacterial viability was assessed by determining total viable counts (TVCs), (5 × 10^6^ cells ml^− 1^) bacterial cells at stationary phase were cultured in Erlenmeyer flasks containing 20 ml NB supplemented with the extracts SICs. Cultures were incubated at 37 °C and 100 rpm for 8 h. At 60 min intervals, samples were collected, diluted using Ringer’s solution 0.25% (Oxoid, UK), cultured on NA plates and incubated at 37 °C for 24 h. The number of cells surviving treatments was estimated. Three biological replicates were measured in distinct instances, and the standard errors were calculated.

### Azocasein degrading proteolytic activity

To measure the proteolytic activity of the tested pathogen, azocasein assay was used as described previously by Kessler et al. [25]. 150 μl of treated (with the SICs of gall extracts) and untreated cell-free supernatants of *P. aeruginosa* was added to tubes containing 1 ml of 0.3% azocasein solution (Sigma, USA) in 0.05 M Tris-hydrochloric acid and 0.5 mM CaCl_2_ (pH 7.5). The suspension was incubated for 15 min at 37 °C. l0% Trichloroacetic acid was used to stop the reaction. The solutions were centrifuged, and the absorbances at 400 nm were detected.

### Protease assay

Briefly, extracts treated and untreated LB agar plates containing 2% skim milk were inoculated distinctly with *P. aeruginosa* and incubated at 37 °C up to 48 h. A clear area surrounded the colonies indicated proteolysis of casein [26].

### Pyocyanin assay

For pyocyanin assessment, overnight cultures in LB broth were standardised to an OD550 of 0.5 and diluted 1:10 in pyocyanin production broth medium (PPB; 2% peptone, 0.3% MgCl_2_, 1% K_2_SO_4_,). Twenty milliliters of the diluted culture treated with the SICs of the gall extracts were grown in PPB at 37 °C for 24 h. The pyocyanin was extracted by three ml of chloroform; the yielded blue-green colour was re-extracted into one ml 0.2 M HCl. The absorbance of the red colour on the top was estimated at 520 nm. The level of pyocyanin in the presence and absence of extracts was calculated by multiplying the absorbances by 17.07 [27].

### Motility assays

The described method by Dezeil et al. [28] was performed to measure the swimming and swarming motilities. Fresh cultures of *P. aeruginosa* initiated from a single colony were point inoculated onto swimming medium (1% tryptone, 0.5% NaCl and 0.3% agar) and swarming agar (1% peptone, 0.5% NaCl, 0.5% agar and 0.5% of filter-sterilized D-glucose) containing SICs of the gall extracts, and incubated at 30 °C for 24 h. The swimming and swarming migration was measured by following the extent zones of the bacterial colony in mm. Swim and swarm agar plates with no extracts were preserved as control.

### Biofilm assay

The impact of *Q. infectoria* gall extracts on the biofilm development was estimated by a microtiter plate (MTP) assay [29]. Overnight cultures of the tested bacterium inoculated into LB broth containing SICs of the *Q. infectoria* gall extracts in flat bottoms polystyrene MTPs (Costar/USA) and incubated aerobically in a static condition for 24 h at 37 °C. Wells without extracts concentration used as control. After incubation, the liquid cultures discarded, the wells were washed three times by phosphate buffer saline and stained with 1% crystal violet (CV) solution. The MTP wells then washed with distilled water, and the biofilm was quantified by eluting the dye with ethanol. The ability of the tested pathogen to adhere to abiotic surfaces was detected by measuring the absorbance at a wavelength of 490 nm by an Elisa reader (Epson, Biotech, UK).

### In situ visualisation of biofilms

To visualise the biofilms, cover glass method was used as described by Al-Shabib et al. [30]. Briefly, 1% of the overnight cultures (OD adjusted to 0.4 at 600 nm) of *P. aeruginosa* was transferred to 1 ml fresh LB medium containing coverslips of 1 cm^2^ in the presence and absence of the gall extracts. After overnight incubation, the coverslips were dipped in distilled water three times to remove the planktonic cells and stained with 1% CV. The biofilms were visualised by the light microscope (MEIJi Techno/ Japan) at a magnification of 40X.

### RNA extraction

To measure the expression of the QS (*las,* and *Rhl*) and *ETA* genes, total RNA was extracted from treated and untreated bacterial cells according to the procedure designated by the manufacturer (Total RNA Purification Kit, Jena Bioscience, Germany). The remaining genomic DNA was removed by RNase-free DNase I (Promega, USA). The purity and the concentration of the extracted RNA were determined by measuring the absorbance at (260/280 nm) by Nanodrop spectrophotometer (IMPLEN).

### Real-time reverse transcription polymerase chain reaction (RT-PCR)

The primer sequences used for quantification of QS and *ETA* genes were as follows (sense and antisense):*lasI,* 5′-ATGATCGTACAAATTGGTCGGC-3′ and 5′- GTCATGAAACCGCCAGTCG-3′ [31]; *lasR*, 5′- ATGGCCTTGGTTGACGGTT-3′ and 5′-CAAGATCAGAGAGTAATAAGACCCA-3′ [32]; *rhlI, 5′-* TTGGTCATGATCGAATTGCTC-3′ and 5′- ACGGCTGACGACCTCACAC-3′; *rhlR*, 5′- CAATGAGGAATGACGGAGGC-3′ and 5′- GCTTCAGATGAGGCCCAGC-3’ [31]; and *ETA,* 5′- GACAACGCCCTCAGCATCACCAGC-3′ and 5′- CGCTGGCCCATTCGCTCCAGCGCT-3’ [33, 34].

One-step quantitative SuPrimeScript RT-PCR kit with SYBER Green l (Genaid, Korea) was used to measure the relative expression. Real-time PCR was performed using PCR^max^ Eco 48 Real-Time PCR system. The reaction process was accomplished following the manufacturer’s conditions in a 20 μl total volume as follows: 50 °C for 20 min (cDNA synthesis), 95 °C for 10 min (initial denaturation), fourty cycles of 95 °C for 15 s (denaturation), and 60 °C for 60 s (annealing /extension). Results were analysed using the ΔΔCt method, and changes in copy number were calculated [35]. The difference in transcripts was considered with respect to control (untreated culture) using the comparative Ct method. Real-time PCR amplifications were conducted in triplicate.

### High- performance liquid chromatography(HPLC)

The plant fractions were dissolved in their respective solvents (ethyl acetate/ n-butanol/ethanol/ water) and were filtered by a polyvinylidene fluoride hydrophilic membrane syringe (0.22 μm, Hinitmoedia), and 10 μL aliquots of the filtrate were injected into the LC-MS/MS system. LC-MS/MS system used for the qualitative and quantitative analysis of 37 phytochemicals consists of Shimadzu Nexera model UHPLC coupled to Shimadzu LCMS 8040 model triple quadruple mass spectrometer. The liquid chromatograph composed of LC-30 AD model gradient pump, DGU-20A3R model degasser, CTO-10ASvp model column oven, and SIL-30 AC model autosampler. The chromatographic partition was achieved on an Inertsil ODS-4 model C_18_ (100 mm × 2,1 mm, 2 μm) column. The column temperature kept at 35 °C during the analysis. The mobile phase comprised of water (A, 10 mM ammonium formate-0.1% formic acid) and methanol (B). The applied gradient profile was optimized as 5–20% B (0–10 min), 20% B (10–22 min), 20–50% B (22–36 min), 95% B (36–40), 5% B (40–50 min). The flow ratio of the mobile phase was 0.25 ml/min, and the injection volume was 4 μl. The optimum ESI parameters for the mass spectrometer were measured as; 350 °C interface temperature, 250 °C DL temperature, 400 °C heat block temperature, 3 l/min and 15 l/min were nebulizer and drying gas (N_2_) flow rates, respectively.

### Statistical analysis

The assay results were analysed using GraphPad Prism 6.0 software. One-way analysis of variance (ANOVA) method was used for multiple comparisons.

## Results

### Inhibition of planktonic *P. aeruginosa* by galls extracts of *Q. infectoria* and growth analysis

**Fig. 1:**
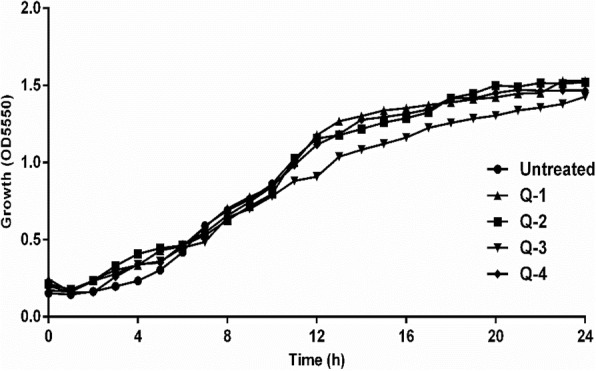
Growth curve of *P. aeruginosa* isolates in the presence of *Q. infectoria* gall extracts over 24 h; points denote means from three biological replicas. Q-1: ethyl acetate, Q-2: n-butanol, Q-3: ethanol, Q-4: aqueous extract

**Fig. 2:**
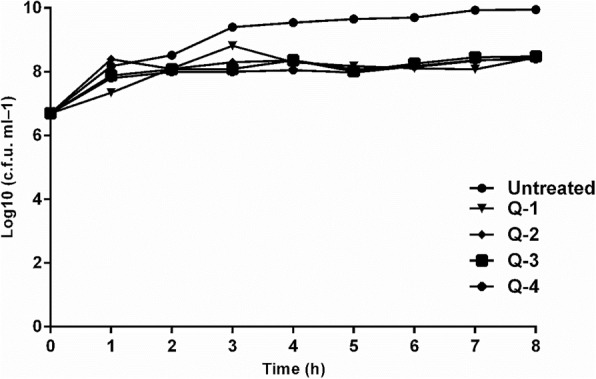
TVCs of *P. aeruginosa* isolates grown in the presence of *Q. infectoria* gall extracts over 8 h; points denote the mean from three biological replicas. Q-1: ethyl acetate, Q-2: n-butanol, Q-3: ethanol, Q-4: aqueous extract. Q-1: ethyl acetate, Q-2: n-butanol, Q-3: ethanol, Q-4: aqueous extract

The MICs of *Q. infectoria* gall extracts were detected to select the SICs to evaluate their influence on the bacterial growth and inhibition of QS-regulated behaviours. The MICs and the MBCs of the extracts against the clinical isolate of *P. aeruginosa* (which resisted all antimicrobials examined in the present work except Amikacin and Ciprofloxacin) were determined and the SICs of the extracts with ethyl acetate (Q-1), n-butanol (Q-2), ethanol (Q-3), water (Q-4) used throughout the study (Table 1). Ethanol fraction recorded the least MIC (2.5 mg/ ml) followed by ethyl acetate (5 mg/ml). Growth curves in Fig. 1 show that the growth proportion and the overall cell number in cultures treated with the SICs of gall extracts were not affected significantly when compared to untreated cells. A slight insignificant drop in the total bacterial cells was observed in extracts treated cultures up to 8 h treatment period when compared to untreated values (Fig. 2).

**Table 1:** Minimum inhibitory concentrations (MICs), Sub-MICs, and Minimum Bactericidal Concentrations (MBCs) of *Q. infectoria* gall extracts of MDR *P. aeruginosa* isolates (mg/ml)

Gall extracts	MIC(mg/ml)	SUB-MIC (mg/ml)	MBC(mg/ml)
Q-1	5	2.5	10
Q-2	10	5	20
Q-3	2.5	1	4
Q-4	20	10	25

*Q-1* Ethyl acetate, *Q-2* n-Butanol, *Q-3* Ethanol, *Q-4* Aqueous extract

### *Q. infectoria* gall extracts impact Qs-regulated virulence factors

The effect of *Q. infectoria* gall extracts on the QS-related virulence was investigated. The ability of the extracted fractions to reduce or prevent the production of the extracellular proteases in *P. aeruginosa* was studied. 7When the bacterium was grown on skim milk plates supplemented with SICs of the extracts, the transparent area surrounding the colonies was reduced significantly or almost abolished entirely as a result of casein break down. Untreated cultures revealed a 25 mm halo, while colonies on extracts plates created smaller (Q-1, Q-3, Q-4) or no halo (Q-2) (Table 2). A significant decrease was also perceived in the total protease activity at the tested concentration. Aqueous and ethyl acetate fractions were more effective than other fractions and significantly reduced the production of protease. Moreover, the extracts were tested to decrease the excretion of QS-dependent pyocyanin, a toxic blue-green substance exclusively produced by *P. aeruginosa* [36]. The data in Table 2 shows the amount of pyocyanin produced by *P. aeruginosa* grown in PPB supplemented with the SICs of the extracts; a significant decrease was detected in the extracts treated cultures. Impact on these distinct QS-regulated virulence factors suggests that compounds from *Quercus* galls affect the regulator genes on the level of expression. This consequence would specify an underlying coordination of gall extracts and the *P.aeruginosa* QS networks.

**Table 2:** Effect of SICs of *Q. infectoria* gall extracts on of virulence factors in *P. aeruginosa*. Data are expressed as mean ± SE .^a^Total protease presented as the absorbance at OD 420, ^b^Exoproteases and ^d^motility (swarming and swimming) data are expressed as the diameter in mm, ^c^Pyocyanin levels were presented as micrograms of pyocyanin produced per microgram of total protein

*P. aeruginosa*	^a^Total protease	^b^Exoprotease	^c^Pyocyanin production	^d^Motility	
				Swarming	Swimming
Untreated	0.706 ± 0.014	25.33 ± 0.88	5.317 ± 0.044	36.7 ± 3.28	54.33 ± 1.202
Q-1	0.24 ± 0.0067	0.0000	0.54 ± 0.023	8.5 ± 0.29	16 ± 0.58
Q-2	0.30 ± 0.0058	17 ± 0.33	0.60 ± 0.012	14 ± 0.60	27 ± 0.23
Q-3	0.31 ± 0.0088	17 ± 0.88	0.55 ± 0.028	15 ± 0.88	23 ± 0.58
Q-4	0.097 ± 0.0033	3.3 ± 0.88	1.5 ± 0.29	6.0 ± 0.58	13 ± 0.88

*Q-1* Ethyl acetate, *Q-2* n-Butanol, *Q-3* Ethanol, *Q-4* Aqueous extract

### Reduction of biofilm formation in clinical isolates of *P. aeruginosa* by *Q. infectoria* gall extracts without affecting the planktonic growth

**Fig. 3:**
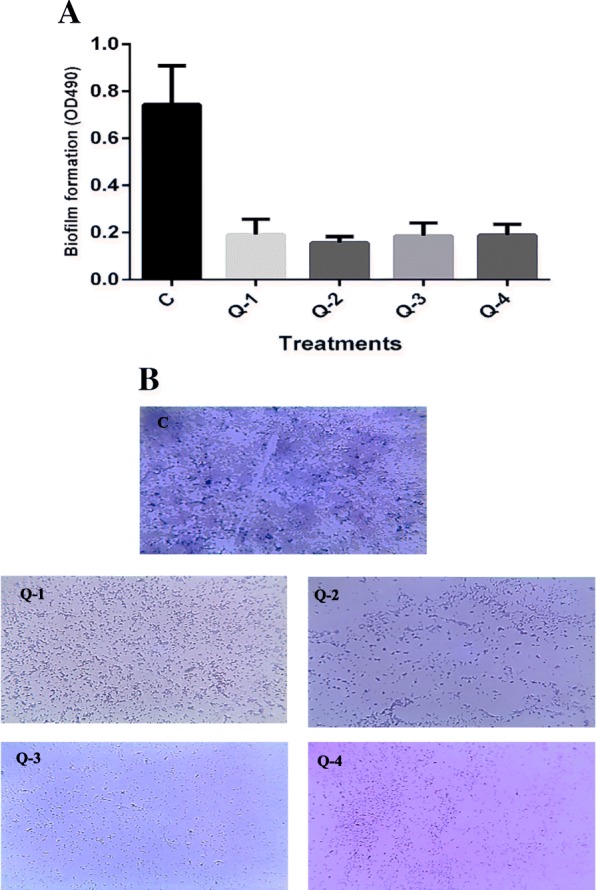
**a** Quantitative measurement of biofilm inhibition in *P. aeruginosa* by crystal violet staining and measuring absorbance at 490 nm. Data are presented as mean ± SE. **b** Crystal violet stained light microscopic images of bacterial biofilms grown in the absence and presence of SICs of *Q. infectoria* gall extracts. **c** untreated, Q-1: ethyl acetate, Q-2: n-butanol, Q-3: ethanol, Q-4: aqueous extract

The ability of *Q. infectoria* gall extracts to decrease or inhibit the development of biofilm was assessed in MTPs of 96-wells. A significant reduction in the biofilm was observed in the studied pathogen when treated with SICs of 2.5, 1, 10 mg/ml for ethyl acetate, ethanol, and aqueous extracts, respectively. Notably, the n-butanol fraction at SICs 5 mg/ml inhibited biofilm formation (Fig. 3a).

The findings of biofilm inhibition consistent positively with swimming and swarming inhibition, a remarkable reduction in both tested motilities was observed mainly in both aqueous and ethyl acetate fractions (Table 2), as bacterial movements have a crucial role in the biofilms adhesion and maturation. It is desired that potential anti-virulence or antibiofilm compounds do not prevent bacterial growth, as this could prevent bacterial resistance.

The analysis of images of light microscopy showed a biofilm with dense layer on the untreated cover glasses, stained and visualised readily under the microscope whereas extracts treated cover glasses revealed a diminishing of biofilm in *P. aeruginosa* isolates (Fig. 3b).

### Repression of quorum sensing genes by *Q. infectoria* gall extracts

#### Expression analysis with RT-PCR

**Fig. 4:**
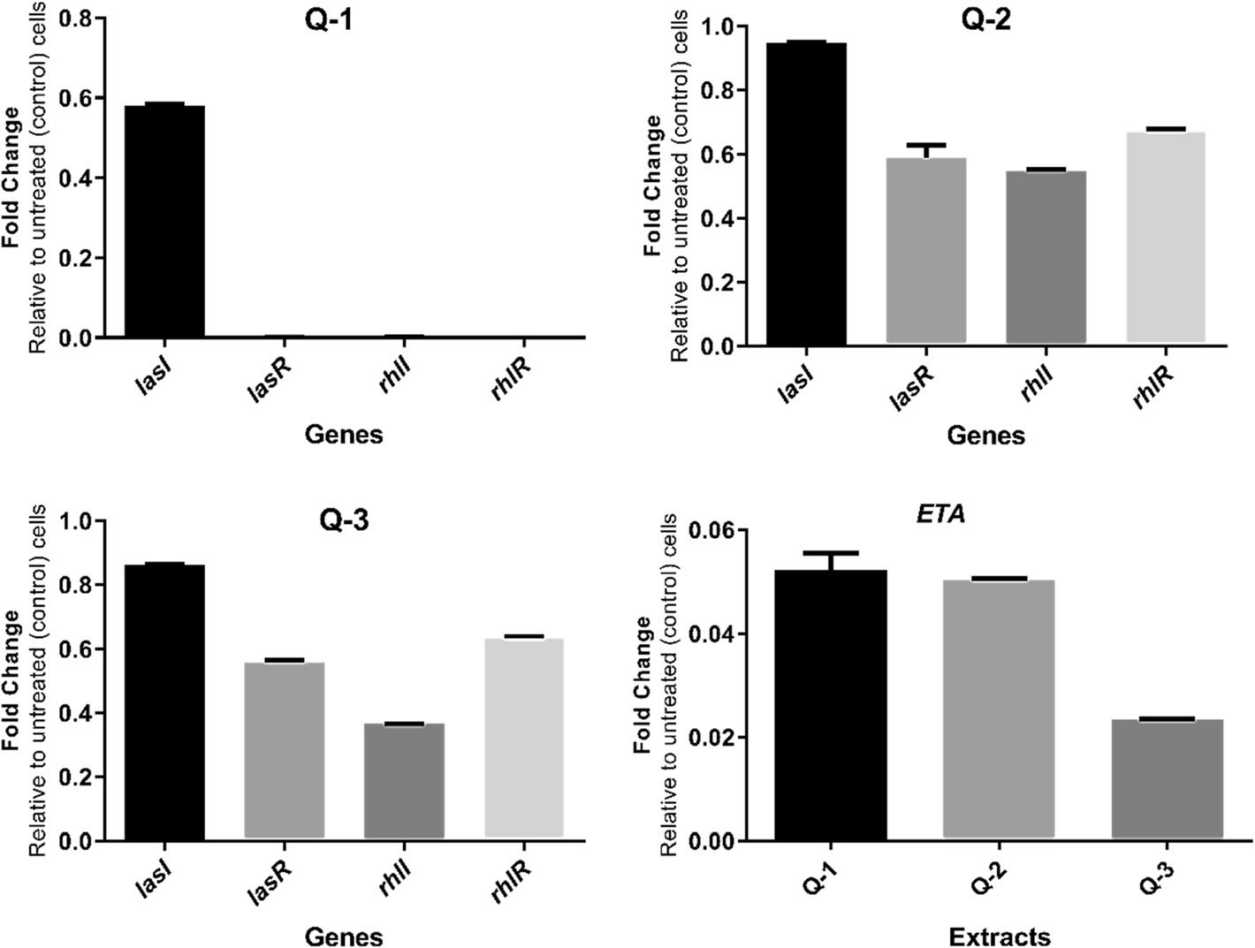
Transcriptional profiles of quorum sensing and *ETA* genes expression of clinical isolates of *P. aeruginosa* when treated with SICs of *Q. infectoria* gall extracts. Transcriptional profiles were measured by RT-PCR. Q-1: ethyl acetate, Q-2: n-butanol, Q-3: ethanol extract, *ETA*: Exotoxin A. The aqueous extract blocked utterly the expression of QS and *ETA* gene (signal not detected)

To study whether the impact of gall extracts on the *P. aeruginosa* virulence was the outcome of inhibition of QS, RT real time-PCR was used to monitor the expression of *lasI*, *lasR*, *rhlI*, and *rhlR* reporter genes. QS networks are among the most attractive non-lethal targets for antimicrobial treatment, as their inhibition would reduce the virulence while conceivably the selection pressure that conventional antimicrobials exert avoided [37]. In this part of the work the inability of *P. aeruginosa* cells to produce autoinducers and exotoxin A following treatment with SICs of *Q. infectoria* gall extracts was tested whether it would target specific genes ((*lasI, lasR, rhlI, rhlR* and *ETA*). The expression of the studied genes was significantly decreased in the extracts treated cells, while the bacterial growth was unaffected (Fig. 4). The most significant decrease was observed in ethyl acetate fraction, while the aqueous extract of the galls completely blocked the expression of all tested genes, this result supported the results of all virulence inhibition in the current study. The observed downregulation of genes and signal molecules combined with impact on virulence factors shows wide-range effects on QS networks.

**Fig. 5:**
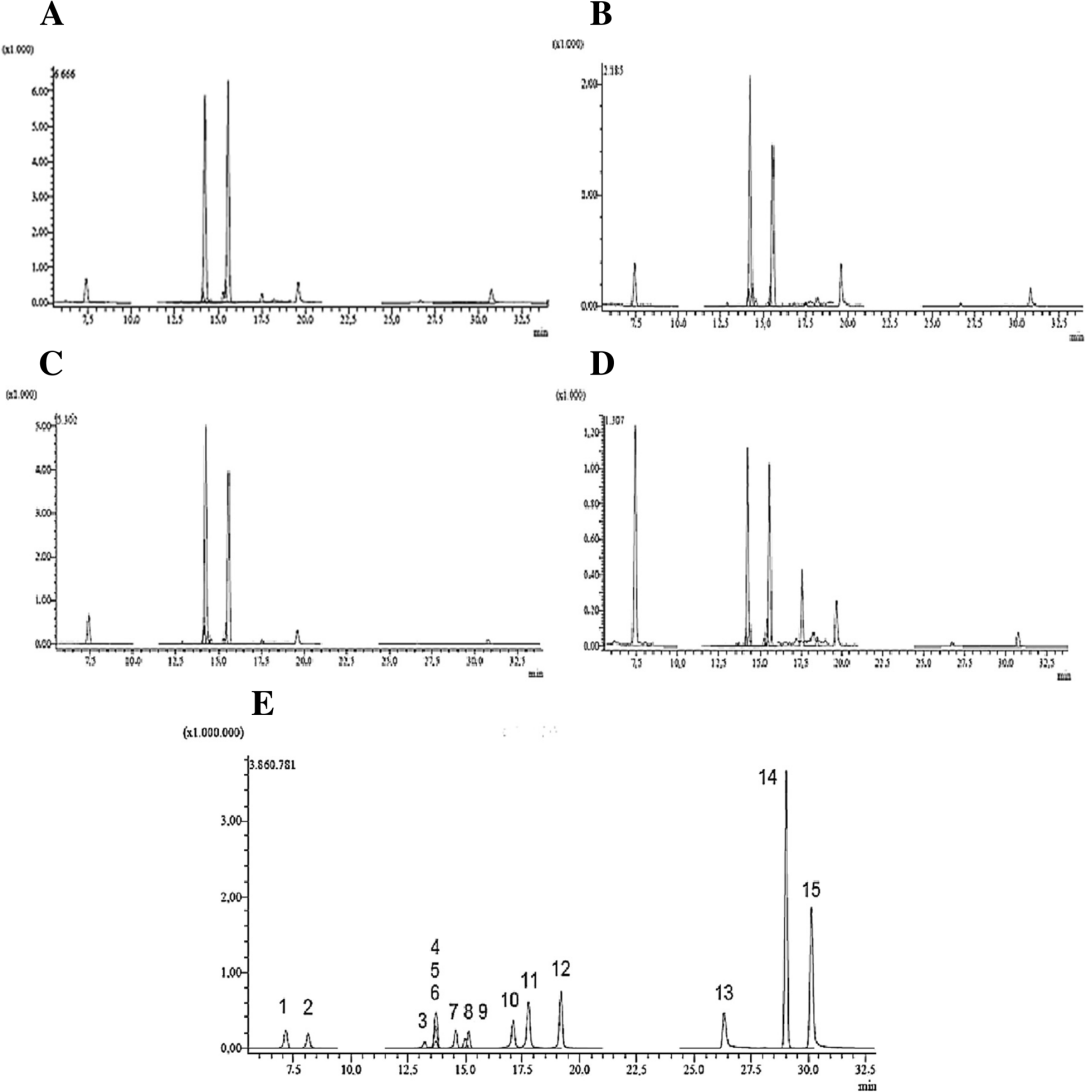
Analytical parameters, identification, and quantification of phytochemicals of *Q. infectoria* gall extracts by LC-MS/MS. **a** ethyl acetate, **b** n-butanol, **c** ethanol, **d** aqueous extract, **e** LC-MS/MS chromatogram of 15 phytochemical standards. 1) Protocatechuic acid, 2) Chlorogenic acid, 3) Luteolin-7-glucoside, 4) Rutin, 5) Hesperidin, 6) Hyperoside, 7) Apigetrin, 8) Quercitrin, 9) Astragalin, 10) Quercetin, 11) Luteolin, 12) Apigenin, 13) Pseudohypericin, 14) Hyperforin, 15) Hypericin

The results of HPLC showed the occurrence of fifteen compounds in the fractions investigated. Two of them of high abundance that were identified as hyperoside and astragalin in all fractions, whereas four major peaks were detected, indicating the abundance in phytochemicals (Fig. 5 and Table 3).

**Table 3:** Analytical parameters of LC-MS/MS method for the analysis of 15 phytochemicals

No.	Analytes	RT^a^	Precursor ion (m/z)^b^	R^2c^	RSD %^d^		Recovery (%)		U^e^
					Intraday	Interdays	Intraday	Interdays	
1	Protocatechuic acid	7.00	153.4	0.9909	0.0060	0.0060	1.0096	0.9988	0.0215
2	Chlorogenic acid	8.03	353.3	0.9910	0.0074	0.0055	0.9941	0.9999	0.0299
3	Luteolin-7-glucoside	13.20	447.0	0.9939	0.0052	0.0037	1.0014	1.0072	0.0086
4	Rutin	13.67	609.1	0.9902	0.0063	0.0070	1.0049	1.0037	0.0136
5	Hesperidin	13.68	611.1	0.9942	0.0081	0.0073	1.0053	0.9994	0.0162
6	Hyperoside	13.69	463.0	0.9905	0.0074	0.0056	1.0039	1.0015	0.0126
7	Apigetrin	14.54	431.0	0.9902	0.0047	0.0067	1.0060	1.0047	0.0132
8	Quercitrin	14.98	447.0	0.9918	0.0079	0.0063	0.9999	1.0002	0.0133
9	Astragalin	15.13	447.0	0.9900	0.0086	0.0077	1.0002	1.0017	0.0153
10	Quercetin	17.10	301.2	0.9962	0.0177	0.0227	1.0010	1.0012	0.0573
11	Luteolin	17.78	285.2	0.9901	0.0119	0.0079	0.9961	1.0007	0.0188
12	Apigenin	19.20	269.2	0.9910	0.0087	0.0090	0.9985	1.0022	0.0181
13	Pseudohypericin	26.34	519.0	0.9908	0.0061	0.0089	1.0033	1.0034	0.0172
14	Hyperforin	28.97	535.3	0.9901	0.0218	0.0164	1.0076	1.0061	0.0418
15	Hypericin	30.18	503.0	0.9925	0.0093	0.0095	1.0104	1.0034	0.0189

^a^*RT* Retention time

^b^*Precursor ion(m/z)* Molecular ions of standard compounds

^c^*R2* Correlation coefficient

^d^*RSD* Relative standard deviation

^e^*U (%)* Percent relative uncertainty at 95% confidence level (k = 2)

## Discussion

The emergence of MDR organisms has driven researches for attenuating virulence via quorum sensing modulation tactics instead of traditional antimicrobial treatments [8].

Infections with *P. aeruginosa* have become a real concern in nosocomial infections, particularly in immunocompromised patients [38]. This pathogen has been listed among the global priority pathogens by WHO [39]. Therefore, to develop unique anti-infectives, a vast number of approaches is currently pursued.

New strategies focus on the effect of plants extracts on the bacterial virulence, more directly, the impact on particular virulence genes is of interest research to fight microbes which resist traditional medications. An effective approach to regulate the production of bacterial virulence and biofilms is through attenuating bacterial QS signalling systems by QS-targeted agents. This unique nonantibiotic treatment can decrease the expression of pathogenic genes, prevent infections, and consequently, the risk of bacterial resistance cells are decreased that has been widely exploited recently [40].

This study characterises *Q. infectoria* gall extracts’ ability to restrict bacterial virulence and their interaction with the bacterial signalling system, using SICs to affect living bacterial cells systematically. Our strategy extensively differs from the use of bactericidal concentrations and illustrates another line of information regarding the *Quercus*’s ability to fight infections. The findings of the present study emphasise that several extracts of *Q. infectoria* galls could decrease or prevent the production of various virulence factors in the clinical isolates of *P. aeruginosa*. It is significantly sustaining that the studied extracts affect MDR isolates at lower levels than recorded for bactericidal concentrations.

To confirm the QS inhibitory, the impact of the galls extracts was evaluated on the QS- related virulence of *P. aeruginosa*. The signalling process in *P. aeruginosa* depends on the AHLs production which involves the las and rhl complexes that regulate the virulence factors such as, pyocyanin, exoproteases, exotoxins and participate in the development of biofilm [8]. *P. aeruginosa* produces exoproteases which contribute significantly in the bacterial pathogenesis, destruct the host tissues and improve the growth and invasiveness of the organism, their synthesis and secretion is a typical cooperative behaviour regulated by QS system [41]. Significant reduction of proteases was indicated in isolates treated with sub-inhibitory concentrations of extracts, particularly aqueous and ethyl acetate extract. Pyocyanin is the secondary metabolite of *P. aeruginosa*, which causes severe toxic effects by degrading the neutrophil-mediated host defence [42]. The significant decrease in the production of pyocyanin is appropriate with a notable reduction in both las and rhl systems expression. Similar results of proteases and pyocyanin inhibition were recorded by Wang et al. [43] who used honey as natural anti-virulence and QS inhibitor. The extracts prohibited the secretion of extracellular proteases differently; ethyl acetate was the most active fraction which wholly eliminated the exoproteases production. Exoproteases are known to be regulated by las operon, which is under the control of lasR and Mvfr systems [28]. Flagella-driven movement is a QS dependent behaviour that has a vital role in the cell/surface attachment during the production of biofilms. Reduction in the swimming and swarming motility is indicative of the reduction in the flagellar synthesis.

Biofilm is a population of microbes attached to surfaces and sustained by the secretion of an adhesive and protective matrix; biofilms are prevalent in nature, medical and other environments. Due to their inherent tolerance and resistance to antimicrobials, they have enormous implications in healthcare-related infections [44]. The biofilms produced by pathogenic bacteria may cause many health complaints in human, like cystic fibrosis, prostatitis, and periodontitis [45]. The cells in the biofilms are known to be more resistant to antimicrobials, and their extinguishing from infected individuals is often problematic. The formation of biofilm in *P. aeruginosa* is controlled by numerous mechanisms, and the main regulating mechanism is the bacterial signalling system. This study demonstrated that *Q. infectoria* galls extracts at SICs could diminish biofilm development and the expression of QS and virulence genes without influencing the growth. Our findings on the inhibition of biofilms by natural products find support from Hayat et al. [46].

As QS inhibition is broadly recognised as an encouraging implement for the management of *P. aeruginosa* infections, the ability of natural products and non-native correspondents to block AHL/LuxR type receptor binding has been more frequently applied and targeted [47]. Quantitative analysis of gene expression showed that *Q. infectoria* gall extracts at their SICs could reduce or even inhibit the expression of studied QS genes differently*.* A remarkable downregulation in the expression of both *P.aeruginosa* QS (las and rhl) pathways which structure the AHL network was indicated. This emphasised that the extracts affected the *P. aeruginosa* virulence by repression the QS systems activity. Our data are consistent with the results obtained by Adonizio et al. [48]. The rhlR system is allied to *Mvfr,* which controls an additional range of bacterial virulence such as, proteases, biofilm development, and bacterial motility [49].

Some virulence factors enable *P. aeruginosa* to adhere and damage tissues for nutrition supply, dissemination, and to increase the survival rate. Exotoxin A is one of them, which is an active enzyme refers to the mono-ADP-ribosyl transferase family and produced by most clinical strains of *P. aeruginosa* [50]. No previous studies have considered the activity of exotoxin A in the presence of plant extracts substances; it can be demonstrated from the results that SICs of the extracts were able to affect the expression of exotoxin A which is considered the main virulence of pathogenic *P. aeruginosa*. Moreover, all fractions of the galls reduced or inhibited the expression of exotoxin A differently; ethanol fraction was the most active fraction reducing the expression. What is more, the aqueous fraction completely blocked the expression which might be due to the water-soluble compounds besides the higher SICs of the aqueous fraction than the other fractions. This result is somewhat impressive because it is crucial for the toxin to be at a sufficient level in the extracellular space for competent killing [51].Our result of exotoxin A finds support from Ahmed & Salih [52] who recently found that low levels of honey could modulate the exotoxin A expression in *P. aeruginosa*.

Furthermore, if both QS systems (las and rhl) are blocked, *P.aeruginosa* could not be able to reset the production of the QS-related virulence factors; this tactic may competently diminish the production of bacterial virulence and high decease rates related to *P. aeruginosa.* Although the mechanisms of action of the medicinal plants is a composite problematic, an overall reduction in the QS system with each of the investigated fractions was observed. This slightly general effect refers to one of two descriptions: the first is related to the various chemical substances in the plants, which may cause diverse impacts on different facets of the QS systems. The other explanation is that the consequence is probably not directly on the las-rhl systems but might be on the other QS regulators, such as *Vfr* [53] or *GacA* [54].

The ability of most plant extracts to combat infections may be attributed to the secondary metabolite products they produced. For *Q. infectoria*, it is evident that our extracts contain many compounds known for their activity on microorganisms as hyperoside and quercetin [55]. From the distinct patterns of HPLC of the solvents fractions, it is recommended that the fractions comprise various active substances and they probably function with distinct mechanisms. Hence, we do not have appropriate data to determine the modes of quorum quenching, further investigations concerning the impact of purified active compounds from the galls of *Q. infectoria* are recommended. Therefore it might be possible to determine therapeutic molecules and develop the responsible compounds for QS inhibition action.

## Conclusions

The current study designated the direct antibacterial activities of the *Q. infectoria* gall extracts against *P. aeruginosa*, which was confirmed by morphological and structural investigations. Differential gene expression was recorded in response to gall extracts exposure, which exhibited in downregulation of several genes involved in the reduction of quorum sensing, and exotoxin A production in the test organism. The potential ability of *Quercus*’s extract to downregulate the expression of these genes is considered valuable for the prophylactic and therapeutic use of medicinal plants. These results afford the first report that galls extracts of *Q. infectoria* affect *P. aeruginosa* with dual mechanisms which involve the direct growth inhibitory effect and the reduction of expression of some bacterial virulence-regulator genes.

## Data Availability

The datasets used and/or analysed during the current study available from the corresponding author on reasonable request.

## Abbreviations

AHL: N-Acyl homoserine lactone
AIs: Autoinducers
DMSO: Dimethyl sulfoxide
*ETA*: Exotoxin A
HPLC: High performance liquid chromatography
LB: Luria-Bertani
MBC: Minimum bactericidal concentration
MDR: Multidrug-resistant
MIC: Minimum inhibitory concentration
NA: Nutrient agar
NB: Nutrient broth
*P. aeruginosa*: *Pseudomonas aeruginosa*
PBS: Phosphate buffer saline
PPB: Pyocyanin production broth
*Q. infectoria*: *Quercus infectoria*
Q-1: *Quercus infectoria* gall extracted by ethyl acetate
Q-2: *Quercus infectoria* gall extracted by n- butanol
Q-3: *Quercus infectoria* gall extracted by ethanol
Q-4: *Quercus infectoria* gall extracted by water
QS: Quorum sensing
SIC: Sub-inhibitory concentration
TSB: Trypto soy broth
TVC: Total viable count
